# Human Metapneumovirus and Other Respiratory Viral Infections during Pregnancy and Birth, Nepal

**DOI:** 10.3201/eid2308.161358

**Published:** 2017-08

**Authors:** Jennifer L. Lenahan, Janet A. Englund, Joanne Katz, Jane Kuypers, Anna Wald, Amalia Magaret, James M. Tielsch, Subarna K. Khatry, Stephen C. LeClerq, Laxman Shrestha, Mark C. Steinhoff, Helen Y. Chu

**Affiliations:** University of Washington, Seattle, Washington, USA (J.L. Lenahan, J.A. Englund, J. Kuypers, A. Wald, A. Magaret, H.Y. Chu);; Seattle Children’s Hospital, Seattle (J.L. Lenahan, J.A. Englund, H.Y. Chu);; Johns Hopkins University Bloomberg School of Public Health, Baltimore, Maryland, USA (J. Katz, S.C. LeClerq);; George Washington University, Washington, DC, USA (J.M. Tielsch);; Nepal Nutritional Intervention Project, Kathmandu, Nepal (S.K. Khatry, S.C. LeClerq);; Institute of Medicine, Tribhuvan University Teaching Hospital, Kathmandu (L. Shrestha);; Cincinnati Children's Hospital Medical Center, Cincinnati, Ohio, USA (M.C. Steinhoff)

**Keywords:** human metapneumovirus, respiratory infections, pregnancy, birth, fever, cough, influenza, vaccines, Nepal, viruses

## Abstract

Human metapneumovirus (HMPV) is a respiratory virus that can cause severe lower respiratory tract disease and even death, primarily in young children. The incidence and characteristics of HMPV have not been well described in pregnant women. As part of a trial of maternal influenza immunization in rural southern Nepal, we conducted prospective, longitudinal, home-based active surveillance for febrile respiratory illness during pregnancy through 6 months postpartum. During 2011–2014, HMPV was detected in 55 of 3,693 women (16.4 cases/1,000 person-years). Twenty-five women were infected with HMPV during pregnancy, compared with 98 pregnant women who contracted rhinovirus and 7 who contracted respiratory syncytial virus. Women with HMPV during pregnancy had an increased risk of giving birth to infants who were small for gestational age. An intervention to reduce HMPV febrile respiratory illness in pregnant women may have the potential to decrease risk of adverse birth outcomes in developing countries.

## Abstract 2

Women infected with human metapneumovirus during pregnancy had an increased risk of delivering infants who were small for gestational age.

Human metapneumovirus (HMPV), a paramyxovirus discovered in 2001, is a previously unrecognized cause of respiratory infections in infants, children, and adults (*1*). HMPV is estimated to cause 5%–25% of respiratory infections among infants and children (*2**–**5*) and 1.5%–10.5% of respiratory infections among adults (*6**–**11*). In children, HMPV is responsible for 5%–15% of hospitalizations for lower respiratory tract illness (*12*). In adult populations, HMPV is responsible for up to 11% of hospitalizations for acute respiratory illness, particularly in adults with underlying heart or lung disease (*5**,**10**,**11*).

Illness and death from respiratory viruses among pregnant women have received greater appreciation globally following the pandemic influenza outbreak in 2009. Pregnancy is known to have an immunomodulating effect, and pregnant women are at elevated risk for complications of both seasonal and pandemic influenza (*13**,**14*). The epidemiology and clinical presentation of noninfluenza respiratory viral infections, including HMPV, during pregnancy, as well as the consequence of such infections on the fetus, are not well described, despite advances in sensitive and rapid molecular diagnostic methods and increasing surveillance of respiratory viruses in diverse populations.

This study describes the clinical characteristics of infection due to HMPV and other respiratory viruses among pregnant women in a rural population in South Asia. It also examines the effects of HMPV infection during pregnancy on birth outcomes.

## Methods

We obtained data for this study during 2 consecutive community-based randomized controlled trials of year-round seasonal influenza vaccination among pregnant women in a rural population in Sarlahi, Nepal. We conducted an active, home-based surveillance with a door-to-door census to identify married women of reproductive age during April 2011–September 2013 (*15*). Follow-up occurred every 5 weeks at households where women of reproductive age resided to determine whether a woman had become pregnant. All married women 15–40 years of age identified as pregnant with gestational age of 17–34 weeks during the study period were offered enrollment and randomized into 1 of 2 study arms: vaccination or placebo.

From the time of vaccination with study vaccine or placebo through 180 days postpartum, a field interviewer visited the household weekly to conduct a morbidity interview for each day in the preceding week. If a participant had an influenza-like illness (ILI) episode (defined as reported fever plus >1 of the following symptoms: cough, myalgia, rhinorrhea, or sore throat), a midnasal swab specimen was collected and tested for respiratory viral infection by real-time reverse transcription PCR (*16**–**19*). Viral infection was defined as the molecular detection of the virus concurrent with symptoms of a respiratory illness. Any symptoms separated from the illness episode by at least 7 symptom-free days were considered part of a separate illness episode. This analysis does not include influenza, however, because it is analyzed separately as part of the clinical trial of maternal immunization (*20*).

We estimated gestational age at time of respiratory virus infection in pregnancy by subtracting the date of last menstrual period from the date of delivery, based on maternal recall. The “mother’s smoking” variable captured whether cigarettes or bidi (a hand-rolled cigarette common in South Asia) were smoked in the previous 30 days. Ethnic group was dichotomized as Pahadi (origins in the hills of Nepal) versus Madheshi (origin in the plains of Nepal). We placed women in the categories of Brahmin (highest caste), Chhetri (higher caste), Vaiysha (working caste), Shudra (lower caste), and Muslim. We defined household size as number of persons sharing a cookstove; we defined household density as the number of persons per room, excluding kitchen and storerooms.

We calculated the incidence of infection by using days of follow-up from enrollment through 180 days postpartum. Among women with HMPV and other respiratory viruses, the duration of illness and the week following the illness period were excluded from time at risk. Descriptive statistics were used to summarize the characteristics of pregnant and postpartum women with and without HMPV and other respiratory viral infections. Bivariate Poisson regression analyses were performed to assess potential risk factors including household density, number of children <5 years of age in the household, caste, ethnic group, maternal education, and smoking.

To assess differential clinical symptoms related to infection comparing pregnant and postpartum women, we assigned a 1-point score to each of the following symptoms: fever, cough, sore throat, rhinorrhea/nasal congestion, and myalgia (*21**–**23*). We used a Wilcoxon rank-sum test to compare severity scores and total days with symptoms among pregnant and postpartum women. We also compared the proportion of women experiencing each individual symptom for pregnant and postpartum women.

Birth outcomes assessed were birthweight and gestational age at birth. We defined low birthweight (LBW) as <2,500 g. We defined small for gestational age (SGA) with INTERGROWTH-21 standards (*24*). We defined preterm birth (PTB) as birth before 37 weeks completed gestation. We assessed associations between HMPV during pregnancy and birthweight and gestational age with linear regression, whereas we used Poisson regression to assess binary outcomes (LBW, SGA, PTB). As a reference, we also compared birth outcomes between women with and without any febrile respiratory illness during pregnancy. Finally, we calculated the proportion of ILI episodes with respiratory viruses other than HMPV; the symptoms associated with these episodes are reported.

We performed analyses using SAS version 9.4 (SAS Institute, Cary, NC, USA) and Stata version 13.1 (StataCorp LLP, College Station, TX, USA). Institutional review board approval for the randomized controlled trial was given by the Johns Hopkins University Bloomberg School of Public Health, Cincinnati Children’s Hospital, the Institute of Medicine at Tribhuvan University, and the Nepal Health Research Council, with deferral from Seattle Children’s Hospital. Approval for this analysis was received from the University of Washington institutional review board. The primary trial was registered under ClinicalTrials.gov NCT01034254.

## Results

### Incidence and Risk Factors

During April 2011–September 2013, we enrolled and vaccinated 3,693 eligible women; weekly surveillance visits continued through May 2014. Median follow-up time was 48 weeks (interquartile range [IQR] 44–53 weeks) among women with HMPV and 49 weeks (IQR 43–55 weeks) among women without HMPV. Overall, we collected 944 nasal swab specimens from enrolled women.

**Figure 1:**
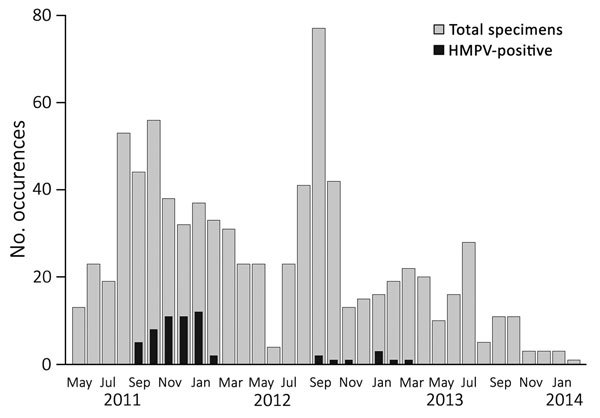
HMPV positivity and seasonality among pregnant women in study of HMPV and pregnancy, Sarlahi, Nepal, April 2011–September 2013. Women were followed until 180 days after birth. HMPV, human metapneumovirus.

During this 3-year period, 55 (1.5%) women had an HMPV illness episode. The overall incidence of HMPV was 16.4 cases/1,000 person-years (95% CI 8.9–30.3); this represents 25 cases among pregnant women (16.7 cases/1,000 person-years, 95% CI 10.8–25.8) and 30 cases among postpartum women (16.1 cases/1,000 person-years, 95% CI 7.5–30.1). Incidence peaked at 71.9 cases/1,000 person-years for September 2011–January 2012. During May 2013–January 2014, no HMPV infections were observed (Figure 1). Other respiratory viruses detected among pregnant women in this cohort were rhinovirus (n = 98), coronavirus (n = 30), parainfluenza (n = 23), bocavirus (n = 9), respiratory syncytial virus (RSV) (n = 7), and adenovirus (n = 6) (Table 1).

**Table 1:** Etiology of febrile respiratory illness among pregnant women in Sarlahi, Nepal, April 2011–September 2013*

Type	No. infections	No. co-infections	Frequency of symptoms, no. (%) patients				Sought care/MD, no. (%)†	Median duration of symptoms, d (range)			Severity score
			Cough	ST	RN	Myalgia		Fever	Cough	Any	
Adenovirus	6	2	2 (33)	4 (67)	4 (67)	3 (50)	2 (33)/1 (17)	4 (2–6)	0 (0–4)	5 (4–15)	10 (6–22)
Bocavirus	9	5	8 (89)	6 (67)	7 (78)	5 (56)	5 (56)/0	3 (1–7)	5 (0–11)	7 (3–38)	22 (6–50)
Coronavirus	30	7	21 (70)	17 (57)	19 (63)	16 (53)	17 (57)/1 (3)	2 (1–7)	2 (0–11)	5 (1–67)	11 (2–86)
HMPV	25	11	19 (70)	12 (44)	17 (63)	14 (52)	12 (48)/1 (4)	3 (1–7)	3 (0–26)	5 (2–50)	13 (3–61)
PIV1	4	0	2 (50)	2 (50)	3 (75)	2 (50)	1 (25)/0	4 (2–6)	2 (0–7)	5 (3–13)	16 (5–23)
PIV2	9	4	7 (78)	5 (56)	8 (89)	5 (56)	4 (44)/0	2 (1–7)	2 (0–6)	7 (2–18)	16 (5–27)
PIV3	7	2	4 (57)	4 (57)	6 (86)	5 (71)	6 (86)/1 (14)	3 (2–6)	3 (0–6)	7 (2–12)	21 (3–31)
PIV4	3	0	3 (100)	2 (67)	3 (100)	1 (33)	2 (67)/1 (33)	2 (2–3)	6 (4–13)	7 (5–13)	15 (10–21)
Rhinovirus	98	13	71 (72)	63 (64)	66 (67)	45 (46)	39 (40)/8 (8)	2 (1–10)	3 (0–25)	5 (1–50)	9 (1–93)
RSV	7	4	5 (71)	3 (43)	6 (86)	4 (57)	4 (57)/1 (14)	2 (1–7)	2 (0–3)	3 (2–18)	9 (4–24)

*No virus was detected in 137 women; no swab was taken from 452 women. Total febrile respiratory illnesses: 767; because of co-infections, the number of total infections is 789. HMPV, human metapneumovirus; PIV, parainfluenza virus; RSV, respiratory syncytial virus; RN, rhinorrhea/nasal congestion; ST, sore throat. †Care means seen for care because of illness; MD means seen by a medical doctor.

Among the 25 pregnant women with HMPV, median gestational age at time of illness was 32.5 weeks (IQR 22.0–37.0 weeks). Women with and without HMPV had similar body mass indexes at enrollment: a median of 20.1 (IQR 18.4–22.4 weeks) among women with HMPV and 20.7 (IQR 19.1–28.0 weeks) among women without HMPV. Hypertension was uncommon in the cohort, identified in 43 (1.2%) of women without HMPV and in no women with HMPV.

Median household density was 3 persons/room among women with and without HMPV. Median years of maternal education was 0 (IQR 0–8.0) among women with HMPV and 5 (IQR 0–10.0) among women without HMPV. Three (5.5%) women with HMPV and 108 (3.1%) women without HMPV had smoked in the 30 days before enrollment (Table 2). In Poisson regression analysis, none of the risk factors analyzed, including caste and household density, altered HMPV risk.

**Table 2:** Demographic characteristics and bivariate RR estimates for HMPV infection among pregnant and postpartum women in Sarlahi, Nepal, April 2011–September 2013*

Characteristic	HMPV-positive, n = 55	HMPV-negative, n = 3,638	RR (95% CI)	p value
Age at enrollment, y, median (IQR)	22 (20–25)	23 (20–26)	1.0 (0.9–1.1)	0.96
Smoking†	3 (5)	108 (3)	0.6 (0.0–37.9)	0.83
BMI at enrollment, median (IQR)	20 (18–22)	21 (19–28)	1.0 (0.8, 1.2)	0.66
Ethnic group				
Pahadi	30 (56)	1,997 (57)	Ref	
Madeshi	24 (44)	1,503 (43)	1.0 (0.4–3.3)	0.89
Caste				
Brahmin	3 (5)	377 (11)	0.5 (0.0–5.4)	0.56
Chhetri	5 (9)	445 (13)	Ref (combined)	
Vaiysha	30 (56)	1,933 (55)		
Shudra	11 (20)	452 (13)		
Muslim	4 (7)	284 (8)		
Household size, median (IQR)				
Children <5 y of age	1 (0–1)	0 (0–1)	1.3 (0.7–2.4)	0.41
Density‡	3 (2–6)	3 (2–4)	1.1 (0.9–1.3)	0.20
Education, y, median (IQR)	0 (0–8)	5 (0–10)	0.9 (0.8–1.1)	0.22
Literacy	25 (50)	1,991 (60)	0.6 (0.2–2.2)	0.46

*Values are no. (%) patients except as indicated. BMI, body mass index; HMPV, human metapneumovirus; IQR, interquartile range: Ref, referent; RR, relative risk. †Defined as smoking at enrollment. ‡Defined as persons/room.

### Clinical Symptoms

**Figure 2:**
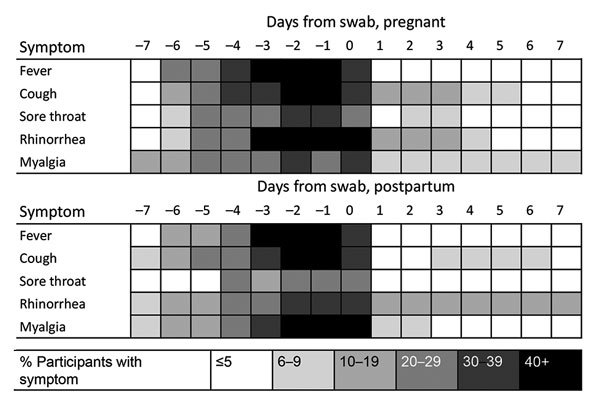
Symptom duration among pregnant and postpartum women in study of human metapneumovirus and pregnancy, Sarlahi, Nepal, April 2011–September 2013.

The most common symptom among pregnant and postpartum participants with HMPV infection was cough (67.3%), followed by rhinorrhea/nasal congestion (58.2%) and myalgia (56.4%). All women with HMPV detected had fever, as this symptom was required for a swab to be taken (Table 3). Median symptom duration was 5 days among both pregnant and postpartum women (Table 3; Figure 2). No difference was noted in the presence or duration of symptoms between pregnant and postpartum women.

**Table 3:** Proportion of pregnant and postpartum women with HMPV infections who had selected symptoms and illness severity, Sarlahi, Nepal, April 2011–September 2013*

Measure	Total, n = 55	Pregnant, n = 25	Postpartum, n = 30	p value
Symptom, no. (%)				
Fever†	55 (100)	25 (100)	30 (100)	NA
Cough	37 (67)	17 (68)	20 (67)	0.92
Sore throat	23 (42)	11 (44)	12 (40)	0.76
Rhinorrhea/nasal congestion	32 (58)	15 (60)	17 (57)	0.80
Myalgia	31 (56)	12 (48)	19 (63)	0.25
Visit for care	27 (49)	12 (48)	18 (60)	0.49
Severity measure, median (range)				
Severity score	10 (1–61)	13 (3–61)	9 (1–38)	0.70
Fever duration, d	3 (1–8)	3 (1–8)	2 (1–8)	0.15
Cough duration, d	2 (0–27)	3 (0–27)	2 (0–10)	0.78
Symptom duration, d	5 (1–31)	5 (2–31)	5 (1–28)	0.36

*NA, not applicable. †Subjective fever required for nasal swab specimen collection.

In respiratory disease associated with respiratory viruses among pregnant women overall, cough was the most common symptom in all illnesses except parainfluenza and RSV, for which rhinorrhea/nasal congestion was the most common symptom. The longest duration of symptoms was observed among women with bocavirus and parainfluenza 2–4 (7 days). The highest severity score was observed among women with bocavirus, who had a score of 22 (range 6–50) (Table 1).

Twenty-four (43.6%) women with HMPV also experienced viral co-infections, with rhinovirus the most common (n = 14; 25.4%), followed by coronavirus (n = 4; 7.3%) and parainfluenza (n = 3; 5.5%). One pregnant woman had HMPV, rhinovirus, and coronavirus concurrently. Symptom duration and severity were similar between women with and without viral co-infection.

**Figure 3:**
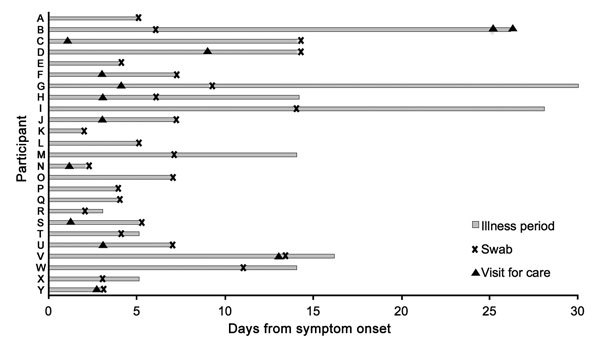
Timing of nasal swab specimen collection and visits for care among 25 pregnant women with human metapneumovirus infection, Sarlahi, Nepal, April 2011–September 2013.

Twelve pregnant women (48.0%) and 18 postpartum women (60.0%) received medical care during the period of their HMPV illness episode (Table 3; Figure 3). Six women (10.9%) sought care >1 time during their illness. Of the 38 total visits, 16 (42.1%) women visited a medicine shop or local doctor, 12 (31.6%) visited a health post, and 10 (24.4%) visited a primary health center, a medicine shop or local doctor, or hospital. Only 1 woman’s hospital visit was potentially related to HMPV. This woman was 4 weeks postpartum at the start of symptoms and had rhinovirus concurrently; she reported 11 days of symptoms, and visited the hospital twice and a health post once during her illness. No participant died of respiratory illness potentially related to HMPV.

### Effect on Birth Outcomes

All women with HMPV during pregnancy delivered live infants. Two (8.0%) of the 25 women with HMPV during pregnancy gave birth to preterm infants; 5 (25.0%) delivered LBW infants. The median gestational age of infants in both groups was 40 weeks (IQR 38–41 weeks), and the median birthweight in both groups was 2.8 kg (IQR 2.4–2.9 kg among women with HMPV, 2.5–3.1 kg among women without HMPV). No differences were noted in birthweight or preterm birth between infants born to women with and without HMPV during pregnancy, nor were differences noted between women with and without fever during pregnancy. However, women with HMPV during pregnancy were found to be 1.7 times as likely to deliver an SGA infant compared with women without HMPV during pregnancy (p = 0.031). Non-HMPV fever during pregnancy did not have a notable effect on SGA risk (Table 4).

**Table 4:** Associations between illness during pregnancy and birth outcomes, Sarlahi, Nepal, April 2011–September 2013 *

Characteristic	No. (%) or median (IQR)				RR (95% CI) or mean difference			
	No fever, n = 3,000	Fever			Any fever	p value	HMPV-positive with fever	p value
		HMPV-negative, n = 668	HMPV-positive, n = 25					
Birthweight, kg	2.8 (2.5–3.1)	2.8 (2.5–3.1)	2.8 (2.4–2.9)		0.0 (−0.1 to 0.1)	0.77	−0.1 (−0.3 to 0.1)	0.23
Low birthweight	542 (25)	132 (26)	5 (26)		1.0 (0.9–1.2)	0.59	1.0 (0.5–2.3)	0.91
Gestational age	40 (38–41)	39 (38–41)	40 (38–41)		−0.2 (−0.3 to 0.1)	0.077	0.2 (−0.7 to –1.1)	0.69
Small for gestational age	830 (38)	198 (39)	12 (63)		1.0 (0.9–1.2)	0.72	1.7 (1.0–2.6)	0.031
Preterm birth	372 (13)	97 (15)	2 (8)		1.2 (0.9–1.4)	0.12	0.5 (0.1–1.9)	0.33

*HMPV, human metapneumovirus; IQR: interquartile range; RR, relative risk.

**Figure 4:**
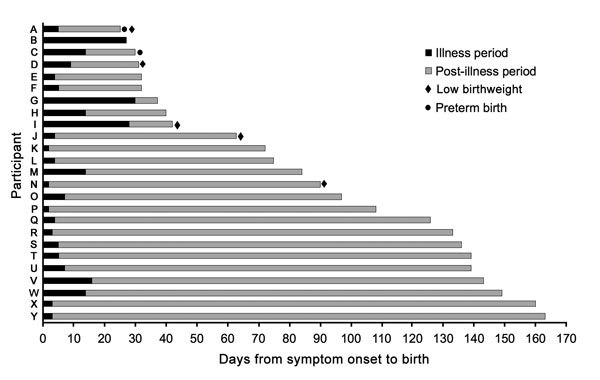
Timing of illness episode and birth outcomes among 25 pregnant women with human metapneumovirus infection, Sarlahi, Nepal, April 2011–September 2013.

Women with HMPV during pregnancy who delivered preterm infants experienced the onset of symptoms 25–30 days before delivery (median 27.5 days); women who delivered LBW infants had symptom onset 25–90 days before delivery (median 42 days) (Figure 4). Three women (60.0%) who delivered LBW infants visited a health post during their illness period; 1 woman (50.0%) who delivered a preterm infant visited a medicine shop/local doctor during her illness.

Among the 30 women with HMPV postpartum, 8 (26.7%) had infants who had HMPV within 1 month of their mother’s illness. Four of these mother–infant pairs had HMPV simultaneously; in 2 pairs, the mother had a positive swab before the infant’s swab, and in 2 pairs the infant had a positive swab before the mother’s swab.

## Discussion

In this analysis, we describe the incidence and clinical presentation of HMPV and other respiratory viruses during pregnancy, as well as birth outcomes associated with HMPV infection during pregnancy. HMPV was a relatively common cause of ILI during and after pregnancy and was associated with an increased risk of SGA birth in women with HMPV respiratory illness compared with women without such illness. HMPV exhibited a clear peak during the first of the 3 years of the study. Despite the rural, resource-limited setting, most women with HMPV accessed healthcare, and nearly 20% visited a health center or physician during their illness.

Incidence of HMPV among women during and after pregnancy was 16.5/1,000 person-years, >4 times as high as the RSV incidence in this cohort during the same time period (3.9/1,000 person-years). Pregnant and postpartum women with HMPV also appeared to exhibit longer fever duration (3 days vs. 2 days) and longer overall symptom duration (5 days vs. 4 days) compared with women in the same cohort with RSV (*25*). Overall, rhinovirus was the most commonly detected pathogen among pregnant women in this cohort (n = 98); coronavirus was also relatively common (n = 30). This finding is notable because Middle East respiratory syndrome coronavirus and severe acute respiratory syndrome coronavirus during pregnancy have been found to be associated with severe complications and illness in the woman and the infant (*26**–**28*).

No notable difference in HMPV severity was noted between pregnant and postpartum women. This finding contrasts with previous literature on influenza and RSV, which demonstrates elevated risk for severe disease among pregnant women (*13**,**14**,**29**–**31*). However, much previous research on respiratory illness severity in pregnancy focused on hospital-related outcomes, such as admission to an intensive care unit, which we were unable to capture in this study.

Previous estimates of adult HMPV incidence ranged from 15 to 53/1,000 person-years (*6**–**11*), but accurate estimates of HMPV incidence are difficult to obtain, particularly in a community setting. Many adults with HMPV infections do not seek medical care, and among those who do, many are not tested for viral infections (*11*). Walsh et al. followed prospectively enrolled young adults (18–40 years of age), healthy elderly adults (≥65 years of age), and high-risk adults (those with symptomatic lung disease) over 4 consecutive winters (*32*). That study found that 4.3%–10.6% of young adults experienced HMPV infection in any given year, higher than the proportion noted in the healthy elderly (2.2%–6.4%) or the high-risk cohort (2.9%–8.6%), supporting our finding that HMPV is a major pathogen among young adults. These findings also align with the proportion of maternal respiratory swabs positive for HMPV in our study (6.7%), although only 55% of participants in the Walsh et al. young adult cohort reported fever (*32*).

The HMPV seasonality demonstrated in this subtropical area is somewhat earlier than the seasonality documented in other settings in the Northern Hemisphere, with most cases typically occurring in January through April (*2**,**4**,**6**,**11**,**32**–**37*). Although most research has been done in temperate climate zones, a study from India demonstrated only 1 HMPV peak over the 3-year study period, which suggests that HMPV may exhibit a nonannual cycle in this setting (*37*).

Although clinic visits in this area of Nepal can be heavily confounded by factors such as location and socioeconomic status, we found that most women with HMPV were seen by a healthcare provider and one quarter of the visits were to a primary health center or MD/MMBS doctor. Considering the frequency of healthcare visits among women with HMPV in this resource-limited region, future research should investigate lower respiratory complications and longer-term sequelae of HMPV in pregnant and postpartum women.

We found that febrile illness due to HMPV during pregnancy is associated with increased SGA risk (risk ratio 1.7; p = 0.031). Infants who are born SGA are at increased risk for poor growth and early death, particularly in developing countries. Reasons for SGA births include maternal malnutrition, use of drugs or alcohol during pregnancy, intrauterine infection, maternal anemia, or hypertension during pregnancy (*38*). Rates of hypertension, maternal smoking, and alcohol use are low in this cohort, and we found no difference in body mass index between pregnant women with and without SGA births. It is possible that a systemic inflammatory response due to HMPV febrile respiratory illness may be associated with increased risk of SGA, although it is not clear why this would be due to HMPV alone rather than febrile respiratory illness in general. No difference was noted in LBW or PTB between infants born to women with and without HMPV during pregnancy. Overall, the observed incidences of LBW and PTB in this cohort were slightly lower than previous estimates in the region, reflecting an overall improvement in health in the population. Among all women enrolled, 25% gave birth to an LBW infant, compared with 30.4% in previous studies; 13% gave birth preterm, compared with 18.3% in previous studies (*15**,**39*). In the parent trial of maternal influenza immunization, influenza vaccination during pregnancy was effective in prevention of influenza infection in infants and was also associated with increased birthweight (*20*). This finding suggests that prevention of potential respiratory illness during pregnancy may decrease the risk of adverse birth outcomes.

Limitations of this study included the relatively small number of women with HMPV during pregnancy. We observed most outcomes at rates at 10%–50%, but with only 25 women having HMPV during pregnancy, we had the power only to detect risk ratios of 1.6 to 3.5. A further limitation was the collection of swabs only in cases of febrile respiratory illness; asymptomatic or afebrile infections would not have been captured. Because previous studies have found that adults with HMPV are often afebrile, with fever reported among 0–55% of adults, we likely underestimated the true incidence of HMPV (*6**,**11**,**32**,**34**,**40*). Preceding respiratory viral infections may increase risk for secondary bacterial infections, such as pneumococcus and staphylococcus. Unfortunately, in this study we did not test for bacterial infections and thus cannot assess whether these had an effect on clinical outcomes. The study was confined to a single rural district, which may have limited the variation in risk factors, such as caste and education. However, this region of Nepal (in the plains along the border with Bihar, India) is broadly representative of South Asia, a region with a population density and birth rate among the highest in the world. Few studies of HMPV have been conducted in these types of settings, particularly among pregnant women, which is notable because the presentation and clinical outcomes of disease may differ in regions of the world with high rates of household density, malnutrition, and indoor air pollution.

In summary, in this prospective study involving active, home-based surveillance in a rural South Asia setting, we found that HMPV is a relatively frequent cause of symptomatic febrile illness during pregnancy. These data may help providers to make decisions about therapeutic care, particularly as more inexpensive viral diagnostic tests become available. Finally, as attempts to develop a vaccine or antiviral therapy for HMPV are currently under way, the identification of risk factors for infection and for severe disease is crucial to identify groups that would benefit most from these advances, and to help develop focused prevention strategies.
